# The Effect of Mechanical Elongation on the Thermal Conductivity of Amorphous and Semicrystalline Thermoplastic Polyimides: Atomistic Simulations

**DOI:** 10.3390/polym15132926

**Published:** 2023-07-01

**Authors:** Victor M. Nazarychev, Sergey V. Lyulin

**Affiliations:** Institute of Macromolecular Compounds, Russian Academy of Sciences, Bolshoi Prospect V.O. 31, 199004 St. Petersburg, Russia; s.v.lyulin@gmail.com

**Keywords:** thermal interface materials, polyimides, thermal conductivity, mechanical elongation, molecular dynamics simulations

## Abstract

Over the past few decades, the enhancement of polymer thermal conductivity has attracted considerable attention in the scientific community due to its potential for the development of new thermal interface materials (TIM) for both electronic and electrical devices. The mechanical elongation of polymers may be considered as an appropriate tool for the improvement of heat transport through polymers without the necessary addition of nanofillers. Polyimides (PIs) in particular have some of the best thermal, dielectric, and mechanical properties, as well as radiation and chemical resistance. They can therefore be used as polymer binders in TIM without compromising their dielectric properties. In the present study, the effects of uniaxial deformation on the thermal conductivity of thermoplastic PIs were examined for the first time using atomistic computer simulations. We believe that this approach will be important for the development of thermal interface materials based on thermoplastic PIs with improved thermal conductivity properties. Current research has focused on the analysis of three thermoplastic PIs: two semicrystalline, namely BPDA-P3 and R-BAPB; and one amorphous, ULTEM^TM^. To evaluate the impact of uniaxial deformation on the thermal conductivity, samples of these PIs were deformed up to 200% at a temperature of 600 K, slightly above the melting temperatures of BPDA-P3 and R-BAPB. The thermal conductivity coefficients of these PIs increased in the glassy state and above the glass transition point. Notably, some improvement in the thermal conductivity of the amorphous polyimide ULTEM^TM^ was achieved. Our study demonstrates that the thermal conductivity coefficient is anisotropic in different directions with respect to the deformation axis and shows a significant increase in both semicrystalline and amorphous PIs in the direction parallel to the deformation. Both types of structural ordering (self-ordering of semicrystalline PI and mechanical elongation) led to the same significant increase in thermal conductivity coefficient.

## 1. Introduction

The development of new thermal interface materials (TIM) with improved thermal conductivity properties is important in order to address various issues in modern electronic and electrical industries. These issues primarily relate to the need to minimize the size of and lighten electronic components [1,2], including computers, consumer devices, telecommunication infrastructure, LED lighting products, renewable energy, automotive engineering, various types of industrial and medical equipment, wireless systems, and solutions for 5G technology [3]. These materials allow for the removal of heat from the heat source, extend the lifecycle of devices, and reduce the cost of maintenance and emergency repair [3,4]. Recently, the use of polymers has become critical in the manufacture of new devices due to their lower specific mass fraction, convenient recycling and usage capabilities, and good dielectric characteristics [1,2]. The thermal conductivity coefficient (*κ*) of polymers, however, is still rather low, lying in the range from ~0.1 to ~0.5 W/m·K [5,6].

In particular, as noted above, the ability not only to remove heat from heat sources but also to preserve excellent dielectric properties is extremely desirable in electronic and electrical industries when producing new thermal interface materials. Among conjugated polymers [7], thermoplastic polyimides (PIs) [8,9,10] can be used for polymer binding, since they are heterocyclic polymers with one of the best thermal and dielectric properties and are characterized by good thermal stability, mechanical strength, and chemical resistance. These polymers are used as films, fibers, nanofibers, membranes, foams, adhesives, and coatings in various industries, including electronics, aerospace, automotives, and medicine. PIs are often used as heat-control coatings, as well as protective layers for electronic equipment [11] due to their low coefficient of thermal expansion. The total global market for polyimides in 2022 was approximately USD 2.31 billion, with the compound annual growth rate (CAGR) predicted to be 7.8% between 2023 and 2030 [12]. The thermal conductivity of PIs is quite low, however, in comparison with that of other polymers [13,14], a fact which significantly hinders the use of these compounds at the heat–dissipation interface in microelectronic devices. Improving the thermal conductivity of PIs is therefore of great importance.

One possible way to improve the thermal conductivity coefficient of polymers [15,16] is the addition of various organic [17,18,19] or inorganic [20,21] nanoparticles with a high thermal conductivity coefficient. However, the desired thermal conductivity is often paired with a growth in electrical conductivity, which ultimately degrades the dielectric properties [22]. For example, the addition of graphene derivatives can improve the thermal conductivity of materials [17,23]; however, since graphene has a high electrical conductivity, this could impair the excellent dielectric characteristics of PIs [22,24]. Metal-based nanoparticles (Al_2_O_3_, Fe_2_O_3_, etc.) [25,26,27] are also widely used to improve the thermal conductivity properties of polymers; nevertheless, similar to graphene derivatives, the incorporation of metal nanofillers into PI also causes a significant deterioration of their dielectric properties. Boron nitride particles and their derivatives [20,28,29,30,31,32] can act in some instances as a substitute for graphene particles to improve the thermal conductivity properties without losing the dielectric properties of PIs. However, the addition of nanofillers to multicomponent systems has many technological problems related to the dispersion of nanoparticles, heat transfer resistance at the nanofiller–polymer interface, and decrease in the fragility of materials, all of which affect the mechanical properties of polymer nanocomposite materials [33]. Therefore, in some instances, it is necessary to change the heat transfer properties of polymers without the addition of nanoparticles [34,35].

On the one hand, an alternative to adding nanoparticles to polymer binders is to achieve structural order in polymer sample. With respect to semicrystalline polymers, an enhancement in the thermal conductivity of these substances can be achieved by isothermal (structural ordering) [36,37] or nonisothermal crystallization [38,39,40]. The thermal conductivity of the ordered polymer chains may depend on the crystallinity, crystallite orientation, and size, as well as the orientation of the polymer chain in the amorphous region [33]. The structural change in the polymer chains induced by the transition from a disordered amorphous state to a partially or fully ordered state could cause an increase in the phonon-free path, thereby reducing the number of phonon-scattering centers [41]. The improvement in the thermal conductivity coefficient of semicrystalline polyethylene (PE) was found to depend on the crystallinity degree of the samples [42]. Ruan et al. [43] studied the thermal conductivity properties of liquid crystal PI films and found that the orientation of polymer chains reduces phonon scattering between polymer chains, which improves the intrinsic thermal conductivity. Kurabayashi et al. [44] investigated the anisotropy of the PI films and established that enhancement in thermal conductivity was in the lateral direction.

On the other hand, stretching the polymer sample might lead [34,45,46] to an improvement not only in thermal conductivity, but also in other properties [22,47,48] of both polymers and nanocomposites on their bases. Using different experimental techniques, Yoon et al. [34] studied the influence of orientation of amorphous BAPP-ODPA PI on the heat transfer properties. They found that oriented samples, even of amorphous PIs, significantly enhanced the thermal conductivity coefficient. The authors suggested that this improvement could be attributed to the orientation of the molecular chain and the appearance of π–π interactions between the aromatic fragments of the PI chains. Lin et al. [48] investigated the influence of draw ratio on the structural and mechanical properties of amorphous PIs and showed that the uniaxial deformation of amorphous PIs leads to a change in their thermophysical properties. Xiang et al. [49] studied amorphous/low-crystallized PI composite fibers and found an increase in the *κ* value in these systems, owing to the orientation of polymer chain and the formation of interchain hydrogen bonds from the wet spinning or low-ratio thermal drawing process.

A significant increase in the thermal conductivity of the drawn PE nanofibers was observed after mechanical deformation was applied [50]. Furthermore, for crystallizable polymers, the additional deformation of ordered samples might further enhance their thermal conductivity [51]. He et al. [52] studied anisotropic thermal transport in crystalline PE. They found that the thermal conductivity increased in the axial direction with an increase in strain, while the thermal conductivity decreased in the radial direction upon deformation. Muthaiah et al. [53] investigated the influence of the strain amplitude on the thermal conductivity coefficient of amorphous PE at different temperatures to understand the behavior of the value of *κ* upon the deformation up to 400% of the strain. Simavilla et al. [54] studied the strain dependence of thermal conductivity for PE and polystyrene (PS) entanglement melts. The strong anisotropy of *κ* values agreed well with the experimental values. Donovan et al. [55] investigated the influence of the additional off-axis strain on the thermal conductivity of polypropylene films using frequency-domain thermoreflectance and molecular dynamics simulations. A significant improvement in the value of *κ* along the deformation direction could be regulated by deformation in the orthogonal direction. Ito et al. [56] found an increase in the thermal conductivity properties of one polymer chain upon strain application. Generally, mechanical stretching is a useful technique for increasing the thermal conductivity of polymers for thermal interface materials, although the degree of improvement could be limited by the intrinsic thermal conductivity of the polymer and may be characterized by some anisotropy relative to the deformation [57,58]. Previously, computer simulations have made it possible to investigate how even small changes in the chemical structure of polymers can influence on the performance properties of thermoplastic PIs [59,60]. However, there is a lack of simulation studies where the influence of mechanical deformation on the thermal conductivity coefficient was performed for both amorphous and semicrystalline PIs, despite the fact that these polymers are very useful for the production of thermal interface materials.

In this study, we investigated the effects of uniaxial stretching on semicrystalline and amorphous thermoplastic polymers. Two semicrystalline polyimides, BPDA-P3 and R-BAPB as well as the amorphous polyimide ULTEM^TM^ were considered. To analyze the effect of uniaxial deformation on the thermal conductivity coefficient of semicrystalline and amorphous PIs, we determined the values of the thermal conductivity coefficient along and perpendicular to the deformation direction. The results were compared with the thermal conductivity properties of unoriented samples. Additionally, with respect to PI BPDA-P3, a comprehensive comparison of the influence of the type of structural ordering on the thermal conductivity was performed. The structural ordering of semicrystalline BPDA-P3, which appeared during uniaxial deformation, was compared with the properties of that PI self-ordered during 30-µs-long molecular dynamics simulations, which corresponded to an isothermal crystallization process. A comparative study of the thermal conductivity of BPDA-P3 polyimide ordered in different ways is extremely important from a computer simulation point of view, as it allows us to evaluate the difference between the thermal conductivity coefficients of two differently ordered samples. Thus, a short simulation of the mechanical stretching of a polymer sample can significantly reduce the simulation time required for the complete self-ordering of the polymer chains of a semicrystalline polyimide if the resulting thermal conductivity is the same as during a long simulation of self-ordering.

## 2. Methodology

### 2.1. Objects of Study

**Figure 1:**
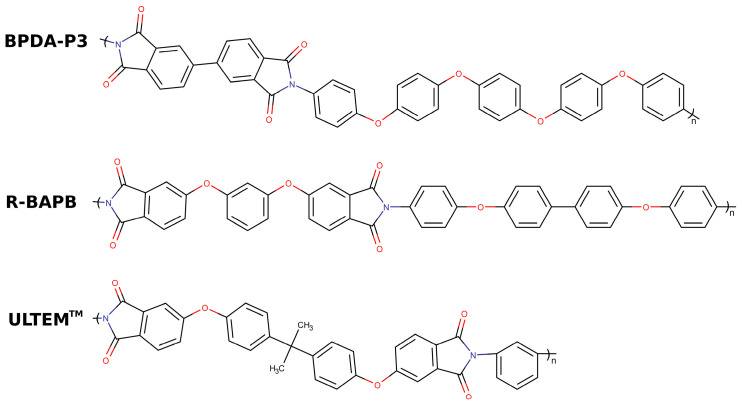
Chemical structure of the repeating unit of the simulated thermoplastic polyimides BPDA-P3, R-BAPB, and ULTEM^TM^.

In this study, the thermoconductive properties were simulated for three thermoplastic polyimides: semicrystalline BPDA-P3 based on 3,3’,4,4’-biphenyltetracarboxylic dianhydride (BPDA) and diamine 1,4-bis [4-(4-aminophenoxy)phenoxy]benzene (P3) [61]; semicrystalline R-BAPB based on 1,3-bis-(3′,4-dicarboxyphenoxy)benzene (dianhydride R) and 4,4′-bis-(4′-aminophenoxy)biphenyl (BAPB diamine) [62,63]; and amorphous polyimide ULTEM^TM^, which is commercially produced by Sabic Innovative Plastics [64] (Figure 1).

### 2.2. Model and Simulation Techniques

In our previous studies [60,65,66,67,68], Gromos53a5 force field [69] was successfully used to simulate the thermophysical, structural, rheological, and mechanical properties of various PIs. The strong orientation of the polymer chains of semicrystalline PIs BPDA-P3 and R-BAPB was observed using atomistic models based on this force field for both unfilled samples [66] and composites reinforced by SWCNT [65,70] or graphene [10,71].

To determine the thermal conductivities of low-molecular-weight paraffin compounds, we developed a universal approach [72] that can also be applied to polymer systems. In this case, molecular samples in a semicrystalline state were created in a natural manner during long-time molecular dynamics (MD) simulations from the amorphous state with the use of Gromacs package [73], which allows calculations on microsecond time scales. Structural ordering of polymer chains is a time-consuming and computationally demanding process. As we have shown [66], the entire unfolding and ordering of polymer chains of an unfilled sample of semicrystalline PI BPDA-P3, even without electrostatic interactions, requires approximately one and a half years of continuous simulation employing 64 processors. To further reduce the required computing power, we used configurations of semicrystalline BPDA-P3 and R-BAPB samples that had previously achieved structural ordering using models based on Gromos53a5 force field.

The LAMMPS MD package [74] is widely used to study the heat transfer properties, since this package implements calculation of the thermal conductivity coefficient by different computational methods. However, since the LAMMPS package is much slower than the Gromacs package, we performed all simulations for the creation of the initial configurations in order to calculate the thermal conductive coefficient in Gromacs. Subsequently, we changed the model to the LAMMPS format.

Several factors determine the need to change the model for thermoplastic PIs. The description of 1–4 van der Waals interactions limits the implementation of the Gromos53a5 force field from the Gromacs package in the LAMMPS package. In the Gromacs package, a list of various scaling parameters for each type of atom is used to describe 1–4 van der Waals interactions; however, in the LAMMPS package, only one scaling parameter for 1–4 van der Waals interactions is used for all types of atoms. Therefore, the previously created ordered configurations of polymer chains in the Gromos53a5 force field were simulated using a model in which valence and nonvalence interactions were described using GAFF [75] force field potentials, with one scaling parameter for 1–4 van der Waals interactions. It should be noted that the GAFF force field used in our previous study [72,76] showed one of the best agreements for calculating the thermal conductivity coefficient of paraffin in the liquid state, as well as good results in simulating the thermal conductive properties of different PIs [77,78,79]. Thus, in this study, a corresponding transition was also made from the Gromos53a5 model to the GAFF model in order to simulate the thermal conductivity of the ordered samples of the PIs under consideration. The procedure to change the model consists of several steps; for more details, see the Supplementary Materials. Furthermore, in the present study, we performed an additional validation of the GAFF force field for the considered systems to simulate their thermophysical properties (See Section 3). Using this force field, computer simulations of the studied PIs could reproduce the experimental value of the thermal conductivity of the ULTEM^TM^ in the glassy state, the experimental ratio between *T_g_* values of the PIs, and the coefficient of thermal expansion (*CTE*) of the considered PIs.

#### 2.2.1. Unordered Polyimide Samples

To accurately verify the use of the GAFF force field for the computer simulation of the thermoconductivity characteristics of thermoplastic PIs, we created and equilibrated thermoplastic PI samples in the GAFF force field. We then studied how MD simulations using PI models based on the GAFF force field could predict their thermodynamic characteristics. To validate the calculation of the partial charge method, which was used to parameterize the electrostatic interactions in the GAFF force field, the partial charges were evaluated using two methods prescribed for the GAFF force field: the semiempirical AM1-BCC approach and *ab initio* calculations HF/6-31G*(RESP) [75,80]. All the bonded and nonbonded parameters (except partial charges) of the GAFF force field were implemented using the ACPYPE program [81]. The AM1-BCC partial charges were calculated using the sqm package in Ambertools [82]. The RESP partial charges were evaluated using the *ab initio* quantum chemistry package Gaussian 09 [83].

As in our previous studies [59,84], each polymer chain has eight repeating units [59]. A polyimide chain with eight repeating units may be considered a Gaussian chain because it has more than 15 Kuhn segments for all studied PIs. Therefore, the polymer chains for BPDA-P3, R-BAPB, and ULTEM^TM^ consisted of 658, 658, and 554 atoms, respectively. MD simulations were carried out using the Gromacs package (v. 2018) [73]. The simulation step was set to 2 fs. The Lennard-Jones potential (potential 6–12) that was truncated at 0.9 nm [75] was employed to simulate van der Waals interactions. The electrostatic interactions were implemented using the particle mesh Ewald algorithm with a cut-off radius of 0.9 nm [85]. The P-LINCS algorithm [86] was used to constrain only the bond lengths between carbon and hydrogen atoms. Simulations were performed using *NPT* ensemble. The Nosé–Hoover thermostat [87] and the Parrinello–Rahman barostat [88] with temperature and pressure coupling times of 1 fs and 5 fs, respectively, were used.

The initial configurations of the PI samples were created from the ‘polymer gas’ according to the methodology developed in our previous study [84]. At 700 K, 27 polymer chains were randomly placed in a periodic box with the side of the box equal to 50 nm. Then, gradual compression was performed at constant pressure of 50 bar for 50 ns. After completion of the compression procedure, the pressure was reduced to 1 bar and each PI sample was equilibrated in the first stage for 3 µs at 700 K. In the second stage, the temperature was immediately increased to 800 K, and then the simulation was performed for 2 µs. Thus, the total equilibration time for each system was 5 µs. During the 5 µs long run, the average sizes of polymer chains were checked. Equilibration was detected by determining the average size of the polymer chains (radius of gyration), which achieved a constant value close to the predicted theoretical value of the size of the polymer molecules in the melt using the free-joint model (Figure S1 in the Supplementary Materials). Throughout the equilibration at 800 K, the translational mobility of the polymer chains was sufficient for the polymer chains to mix well, because the mean squared displacement of the center of mass of the polymer chains was not comparable with the size of the polymer molecule as a whole at 700 K, as shown in Figure S2 in the Supplementary Materials.

#### 2.2.2. Mechanically Elongated Samples of Polyimides

The structural ordering of semicrystalline PIs in simulations is observed even at temperatures slightly higher than the experimental melting temperatures [70,89]. This modeling is physically valid because the simulation results may provide a higher melting temperature of the polymer compared to the experimental findings [90]. Similarity was observed for the increase in glass transition temperature owing to the difference in the time scales of the cooling procedure between the experiment and computer simulation [59]. It should be mentioned that both semicrystalline PIs, BPDA-P3 and R-BAPB, are characterized by experimental values of melting temperatures close to 577 K [61] and 593 K [91], respectively. Therefore, we achieved in simulation precrystalline-oriented structures of samples at a temperature of 600 K.

The unordered PI samples were subjected to uniaxial deformation at 600 K. During the deformation, the PI samples BPDA-P3, R-BAPB, and ULTEM^TM^ were elongated with a strain rate *γ_d_* = 10^−3^ nm/ps (~1.8 × 10^8^ s^−1^) in the *x* direction, similar to [92]. The stretching duration was selected such that the sample size increased by 200% [93] over the original size, representing a threefold increase.

In order (i) to compare the results of the thermal conductivity calculation of the semicrystalline polyimides BPDA-P3 and R-BAPB ordered by mechanical deformation, as well as (ii) to study the difference between the thermal conductivity coefficients of the deformed BPDA-P3 and the self-ordered BPDA-P3 during 30 µs long computer simulations [66], the previously equilibrated samples of BPDA-P3 and R-BAPB using the model based on Gromos53a5 force field were deformed to create mechanically ordered samples [60]. For the amorphous polyimide ULTEM^TM^, the unordered samples created by employing a model based initially on the GAFF force field were elongated at 600 K. The deformed samples of considered PIs were then further simulated at 600 K (models based on the Gromos53a5 force field were used for BPDA-P3 and R-BAPB; model based on the GAFF force field was used for ULTEM^TM^). During these additional simulations, the polyimide chains of BPDA-P3 unfolded and aligned relative to each other, while the PI chains of R-BAPB and ULTEM^TM^ remained practically unchanged (Figure S3 in the Supplementary Materials).

**Figure 2:**
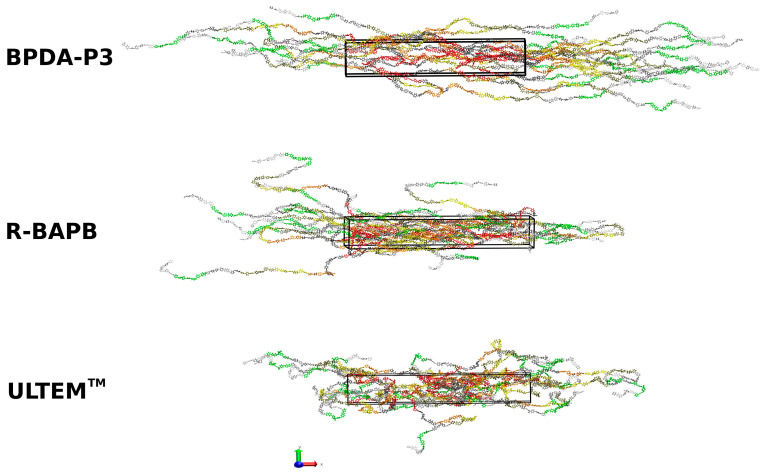
Snapshots of instant configurations of thermoplastic PIs: BPDA-P3 (**top**), R-BAPB (**middle**), and ULTEM^TM^ (**bottom**), which were used for the thermal conductivity calculation after additional simulation following sample elongation.

To estimate the degree of elongation during and after deformation, we calculated the nematic order parameter *S_N_* of the end-to-end vector of the polymer molecule. *S_N_* is determined as the largest eigenvalue of the nematic order tensor $S_{\alpha \beta }=1/N_{ch}\overset{N_{ch}}{\underset{i=1}{\sum }}\frac{3}{2}{\vec{u}}_{i\propto }{\vec{u}}_{i\beta }-\frac{1}{2}\delta_{\alpha \beta },$ where *N_ch_* is the number of chains, $\vec{u}$ is a unit vector parallel to the end-to-end vector of the *i*-th chain, *δ* is the Kronecker delta, and *α*, *β* = x, y, or z are coordinates [70]. Calculated <*S_N_*> mean value of nematic order parameter after additional simulation following sample deformation for semicrystalline BPDA-P3 was 0.96, for semicrystalline R-BAPB was 0.62, and for amorphous ULTEM^TM^ was 0.59 (Figure S3 in the Supplementary Materials). Snapshots of the instance configurations of some PI samples are shown in Figure 2.

#### 2.2.3. Cooling down of Polyimide Samples from the Melt to the Glassy State

To study the thermal conductivity coefficients of thermoplastic PIs for unordered and ordered samples in glassy and melt states, simulations were carried out at temperatures well below (*T* = 290 K), very close to (*T* = 600 K), and much higher (*T* = 800 K) than the melting temperature. Further, cooling from two different temperatures (*T* = 600 and *T* = 800 K) above the experimental melting points of BPDA-P3 and R-BAPB will allow us to trace how the thermal history influences on the value of the thermal conductivity coefficient in the glassy state at room temperature in simulations. The cooling from higher temperatures (*T* = 800 K) may reduce the value of thermal conductivity coefficient in a glassy state, owing to the effect of high temperatures on the orientation of the polymer chains. This suggestion will be investigated further in this study.

In order to investigate the influence of thermal history on the thermal conductivity coefficient, instantaneous heating was performed from *T* = 600 K to *T* = 800 K to achieve the highest considered temperature. Then, all samples were simulated for 10 ns at *T* = 800 K and after cooled from *T* = 800 K to *T* = 290 K at a cooling rate *γ_c_* = 1.5 × 10^11^ K/min. The chosen cooling rate was previously used to study various properties of thermoplastic PIs. We previously showed [59,92] that cooling at this rate is sufficient to model the properties of thermoplastic PIs to predict the experimental relationships of thermophysical, mechanical, and other properties.

As discussed earlier, before the cooling procedure, for the BPDA-P3 and R-BAPB polyimide samples ordered during mechanical stretching and self-ordered BPDA-P3 polyimide samples during a long computer simulation, the Gromos53a5 force-field-based model was changed to the GAFF force field model. With respect to ordered systems, the samples were cooled from *T* = 600 K (the temperature at which ordering was achieved) and from *T* = 800 K. When cooling started at *T* = 600 K, the samples were cooled stepwise from this temperature to *T* = 290 K with a cooling rate *γ_c_* = 1.5 × 10^11^ K/min. While cooling began at *T* = 800 K, for the unordered samples, instantaneous heating was performed from *T* = 600 K to *T* = 800 K, after which the samples were simulated for 10 ns and subsequently cooled stepwise from *T* = 800 K to *T* = 290 K at the same cooling rate.

#### 2.2.4. Calculation of the Thermal Conductivity Coefficient

First, we should know how the method for calculating the thermal conductivity coefficient influences on the simulation results. Thus, to calculate the thermal conductivity coefficient, equilibrium (EMD) and non-equilibrium (NEMD) molecular dynamics simulations were performed. Previously [72], when studying the properties of paraffins, it was found that the EMD method better reproduced the thermal conductivity coefficient of n-eicosane in the crystalline and liquid states than NEMD. Nevertheless, in this study, we also verified that EMD and NEMD can be used to predict heat transfer properties. The parameters of the computer simulation for the PI in LAMMPS (15 April 2020) were similar to those used in our previous study [72].

In the EMD simulations, the Green–Kubo relation was employed to determine the thermal conductivity coefficient *κ*. The calculation of *κ* value was derived from the total heat flux vector $\vec{j}$, as reported in ref. [94]. The autocorrelation function of heat flow, denoted as $\langle \vec{j}\left(0\right)\vec{j}\left(t\right)\rangle$, was integrated to provide the calculation of *κ* value. The resulting equation for the thermal conductivity calculation was as follows: $\kappa =\frac{V}{3k_{B}T^{2}}\int_{0}^{\infty }\langle \vec{j}\left(0\right)\vec{j}\left(t\right)\rangle$ [95,96], where *V* is the volume of a simulation box, *k_B_* is the Boltzmann constant, and *T* is the temperature. The heat flux was determined by 1 ns equilibrium molecular dynamics simulations performed in the microcanonical ensemble (*NVE*). The heat flux was recorded at each 1 fs. We used a correlation time of 10 ps to derive the autocorrelation functions of the heat flux [97]. In order to obtain a reliable estimate of the heat flow, we employed a virial correction to the many-body potentials [97,98]. The calculation was carried out at three distinct temperatures: significantly below (290 K), close to (600 K), and significantly higher (800 K) than the melting temperature. The final value of thermal conductivity coefficient *κ* was computed by taking the mean of the three distinct samples.

The determination of the thermal conductivity coefficient *κ* in NEMD simulations involves the use of a methodology that relies on the creation of a heat flux gradient within the system. The present research employs the reverse non-equilibrium molecular dynamics (r-NEMD) technique, wherein heat flux is produced via the transfer of kinetic energy between atoms located in separate compartments of a simulation box [99,100]. The atoms that were moving at the slowest rate inside the ‘hot’ layers were the ones that participated in an exchange with the atoms that were moving at the fastest rate within the ‘cold’ layers. The frequency at which the interchange of velocities takes place guarantees that the entire kinetic energy of the system is preserved. When establishing a consistent temperature gradient within the given system, the thermal conductivity coefficient *κ* is determined using Fourier’s law, which states that $j_{z}=-\kappa \;\left(dT/dz\right)$. Here, *j_z_* is the heat flow in the defined heat flux direction (such as *z*), and *T* is the temperature.

This methodology for NEMD implemented in LAMMPS was employed to determine the thermal conductivity coefficient *κ*. The simulation box was partitioned into 20 layers [100]. The first and last layers were designated as ‘low-temperature’ layers, while the eleventh layer was designated as a ‘high-temperature’ one. The thermal conductivity of the infinite box was determined by extrapolating the reciprocal of the thermal conductivity (*κ*^−1^) as a function of the reciprocal of the system length (*L*^−1^) when *L*^−1^ = 0 [101,102]. The dimensions of the periodic box were increased two or three times along the coordinate axes relative to their original size. For the enlarged systems, 40 and 60 layers were used. The 21st and 31st layers were correspondingly set as the ‘hot’ layers. The calculation did not incorporate the nonlinear segment of the temperature profile, owing to a significant scattering at the boundaries of the heat source and sink.

Note that the implementation of the SHAKE algorithm for constraining hydrogen bonds during the simulation resulted in significant temperature variation within the NVE ensemble. To mitigate temperature fluctuations in the NEMD simulation, we conducted simulations using the canonical ensemble (*NVT*). As was previously demonstrated [99], the thermal conductivity coefficient exhibited a deviation of no greater than 10%, and our calculations are in agreement with these results.

## 3. Results and Discussion

From the outset, it is crucial to determine which predetermined methods of partial charge calculation, namely AM1-BCC or HF/6-31G* (RESP), are best suited to parameterize the electrostatic interactions of the PI within the GAFF force field. The thermal conductivity coefficients of PIs were analyzed using the EMD and NEMD calculation methods.

### 3.1. Validation of GAFF Force Field for Computer Simulation of Polyimides

Unfortunately, the literature does not provide any available data on the experimental determination of thermal conductivity coefficient *κ* for the PIs BPDA-P3 and R-BAPB. An analysis was conducted to compare the outcomes of a computer simulation with the empirical thermal conductivity coefficient of PI ULTEM^TM^. The *κ* value of the polyimide ULTEM^TM^ provided by the Sabic Innovative Plastics website [64] was selected for comparison with the simulation results. The value of *κ* is 0.220 W/(m·K) at room temperature.

To make a quantitative comparison between the experimental and simulation outcomes of *κ* values, the relative percentage deviation was computed as $\left(\kappa^{sim}-\kappa^{exp}\right)/\kappa^{exp}\times 100\%$ of calculated thermal conductivity values $\kappa^{sim}$ from the experimental thermal conductivity value $\kappa^{exp}$. The results are presented in Table 1.

Based on a comparative analysis of the thermal conductivity coefficient of PI ULTEM^TM^ at room temperature, the following conclusions can be drawn. All computational techniques produced a relative percentage deviation from the experimental value of *κ*, which exceeded 20%.

Overall, the NEMD method, which was worse than EMD, predicted the value of *κ^exp^.* The EMD method yielded the most accurate thermal conductivity results when partial charges were assessed using the HF/6-31G* (RESP) method, with a relative percentage deviation of approximately 20.5% of the calculated $\kappa^{sim}$ value from the experimental $\kappa^{exp}$ value. The EMD method showed better agreement with the experimental thermal conductivity coefficient of PI ULTEM^TM^, which agrees well with the outcome of the study of the thermal conductivity properties of phase-change materials based on paraffin n-eicosane [72].

When using EMD, the AM1-BCC approach exhibited worse results by approximately 9% in terms of the relative percentage deviation compared with HF/6-31G* (RESP). The opposite trend was found for NEMD: the AM1-BCC partial charge calculation method showed an ~18% better reproduction of the experimental value of the ULTEM^TM^ thermal conductivity coefficient than HF/6-31G*(RESP). However, the relative percentage deviation from the experiment for NEMD was much higher than that for EMD. Nevertheless, the disparity in the relative percentage deviation between AM1-BCC and HF/6-31G* (RESP) using EMD is rather low. The increase in the thermal conductivity coefficient in the simulation for both the calculation methods EMD and NEMD, and for the two partial charge calculation methods compared to the experimental value of *κ*, is qualitatively consistent with a small overestimation of the thermal conductivity coefficient, as previously shown for all-atom force fields [72]. This improvement in the thermal conductivity coefficient could be caused by the presence of additional vibrational degrees of freedom for all-atom-based models capable of enhancing phonon transport.

As a result, we conducted further research to determine how these partial charge calculation methods can accurately replicate some of the main thermal–physical characteristics (coefficients of thermal expansion [59] and glass transition temperatures [103]) of the studied PIs. Consequently, as in our previous studies [104,105], (i) the ability of various partial charge parameterization methods to accurately replicate the coefficient of thermal expansion (*CTE*) in the glassy state was studied, and (ii) a comparison was made between the ratio of the glass transition temperatures and the corresponding experimental ratio (Figures S4–S5 and Tables S1–S2 in the Supplementary Materials). The results obtained revealed that the use of the HF/6-31G* (RESP) method reproduces the *CTE* value of PI ULTEM^TM^ in the glassy state better than the AM1-BCC calculation, and the *ab initio* method HF/6-31G* (RESP) could qualitatively reproduce the experimental ratio $T_{g}^{ULTEM^{TM}}>T_{g}^{BPDA-P3}>T_{g}^{R-BAPB}$ between the *T_g_* of the considered PIs. Therefore, the HF/6-31G* (RESP) method is more convenient than the AM1-BCC one to study the influence of ordering on the thermal conductivity coefficient of PI.

The influence of temperature on the thermal conductivity coefficients of PIs was also studied. It was found that an increase in temperature from 290 to 600 K led to an increase in the thermal conductivity of the undeformed samples, and there was a slight decrease in the thermal conductivity coefficient when the temperature rose above the *T_g_* values of the PIs. The results obtained were in good qualitative agreement with the experimental results [106], showing that the thermal conductivity coefficient of amorphous polymers increases with the increasing temperature to the polymer *T_g_*, and a decrease in the value of the thermal conductivity coefficient was observed at temperatures above *T_g_*. The ratio between the thermal conductivity coefficients $\kappa^{BPDA-P3}>\kappa^{R-BAPB}>\kappa^{ULTEM^{TM}}$ of the considered PIs correlates with the ratio between the maximums of the vibrational density of state (*VDOS*) spectra (Figures S6 and S7 in the Supplementary Materials).

It should be noted that for the unordered samples of the considered PIs, cooling from 800 K and 600 K resulted in nearly identical values of the thermal conductivity coefficient at *T* = 290 K. Therefore, for comparison with the results of deformed samples and self-ordering during 30 µs long molecular dynamics simulations, the thermal conductivity coefficients of the unordered samples will be similar at *T* = 290 K for systems cooled from *T* = 800 K and *T* = 600 K to *T* = 290 K.

Thus, the thermal conductivity coefficient *κ* is calculated using the EMD approach and the HF/6-31G*(RESP) method for the partial charge calculation will be used.

### 3.2. Influence of Deformation on the Thermal Conductivity Coefficient

**Figure 3:**
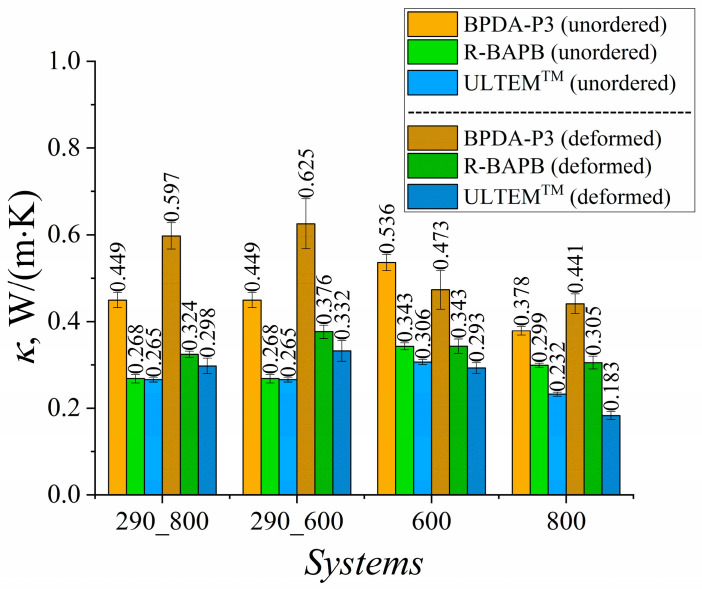
Thermal conductivity coefficients *κ* of BPDA-P3, R-BAPB, and ULTEM^TM^ at different temperatures. Systems ‘290_800’ and ‘290_600’ were simulated at *T* = 290 K. These samples were created by cooling them from *T* = 800 K to *T* = 290 K (‘290_800’) and from *T* = 600 K to *T* = 290 K (‘290_600’). System ‘600’ was simulated at *T* = 600 K, and system ‘800’ was simulated at *T* = 800 K.

To assess the impact of uniaxial deformation on the thermal conductivity of polyimide-based TIMs at various temperatures, the thermal conductivity coefficients were calculated at 290 K, 600 K, and 800 K (Figure 3).

The results showed an increase in the thermal conductivity coefficient *κ* of the deformed samples for semicrystalline BPDA-P3 and R-BAPB, as well as for amorphous ULTEM^TM^ polyimide at *T* = 290 K. The strongest improvement in the heat transfer properties among all the PIs considered was observed for BPDA-P3 (Figure 3). A similar enhancement in the thermal conductivity coefficient of the amorphous PI [34] and PE [54] samples was observed.

**Figure 4:**
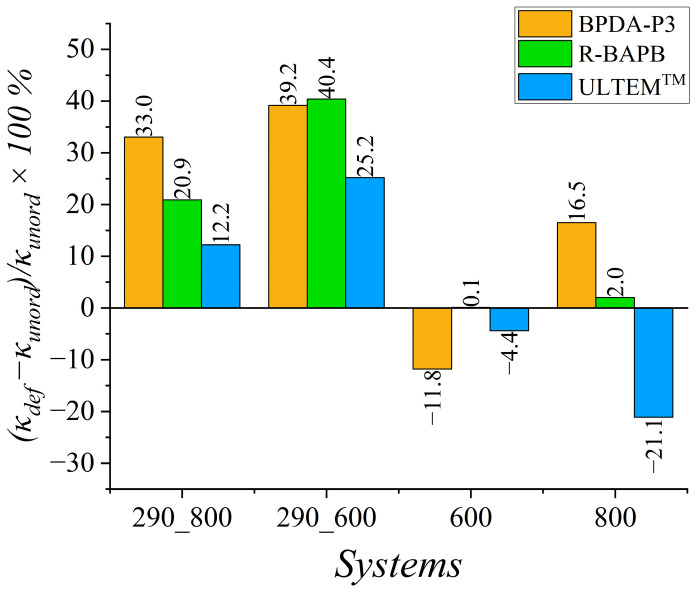
The relative percentage deviation $\left(\kappa_{def}-\kappa_{unord}\right)/\kappa_{unord}\times 100\%$ of the thermal conductivity coefficient *κ_def_* of the deformed samples from the value *κ_unord_* of unordered samples of BPDA-P3, R-BAPB, and ULTEM^TM^ for different temperatures. Systems ‘290_800’ and ‘290_600’ were simulated at *T* = 290 K. These samples were created by cooling them from *T* = 800 K to *T* = 290 K (‘290_800’) and from *T* = 600 K to *T* = 290 K (‘290_600’). System ‘600’ was simulated at *T* = 600 K, and system ‘800’ was simulated at *T* = 800 K.

To estimate the enhancement in the heat transfer properties for mechanically ordered samples, we computed the relative percentage deviation $\frac{\left(\kappa_{def}-\kappa_{unord}\right)}{\kappa_{unord}}\times 100\%$ of the thermal conductivity coefficient value *κ_def_* of the deformed samples from the value *κ_unord_* for the unordered samples, as shown in Figure 4.

Increases in *κ* values of 33% and 20.9% were observed for semicrystalline BPDA-P3 and R-BAPB PIs, respectively, during cooling from 800 K to room temperature. The weakest improvement in the thermal conductivity was observed for the amorphous ULTEM^TM^. This PI demonstrated a relative percentage deviation of 12.2% in *κ* value after deformation.

A much better improvement in the *κ* value for glassy samples at room temperature was observed when cooling started at *T* = 600 K, at which semicrystalline polymers could be partially ordered. The relative percentage deviation almost doubled for semicrystalline R-BAPB and amorphous ULTEM^TM^ PIs compared with the cooling that started at *T* = 800 K. At the high temperatures of 600 K and 800 K, the effect of mechanical orientation on the heat transfer properties deteriorated the thermal conductivity coefficient *κ* of the deformed samples compared to the values for the unordered PIs, as shown in Figure 4. Note that the enhancement in thermal conductivity properties of thermally stable polyimides might improve by only 40% owing to the selected amplitude of mechanical elongation, whereas increasing the strain amplitude might sufficiently change the heat transport properties of amorphous and semicrystalline polymers [53].

**Figure 5:**
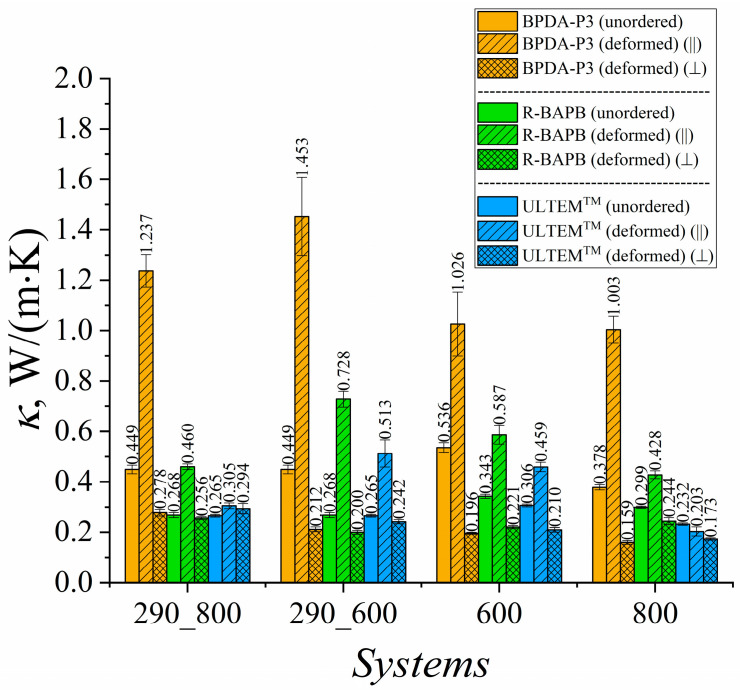
Thermal conductivity coefficients *κ* of polyimides BPDA-P3, R-BAPB, and ULTEM^TM^ at different temperatures. For deformed samples, the thermal conductivity coefficient was evaluated in the directions parallel (||) and perpendicular (⊥) to the deformation direction. Systems ‘290_800’ and ‘290_600’ were simulated at *T* = 290 K. These samples were created by cooling them from *T* = 800 K to *T* = 290 K (‘290_800’) and from *T* = 600 K to *T* = 290 K (‘290_600’). System ‘600’ was simulated at *T* = 600 K, and system ‘800’ was simulated at *T* = 800 K.

The elongation of both semicrystalline and amorphous polymer samples can lead to anisotropy in *κ* value [41]. Furthermore, we estimated the parallel (||) and perpendicular (⊥) counterparts of *κ* value by using the EMD method, as shown in Figure 5.

**Figure 6:**
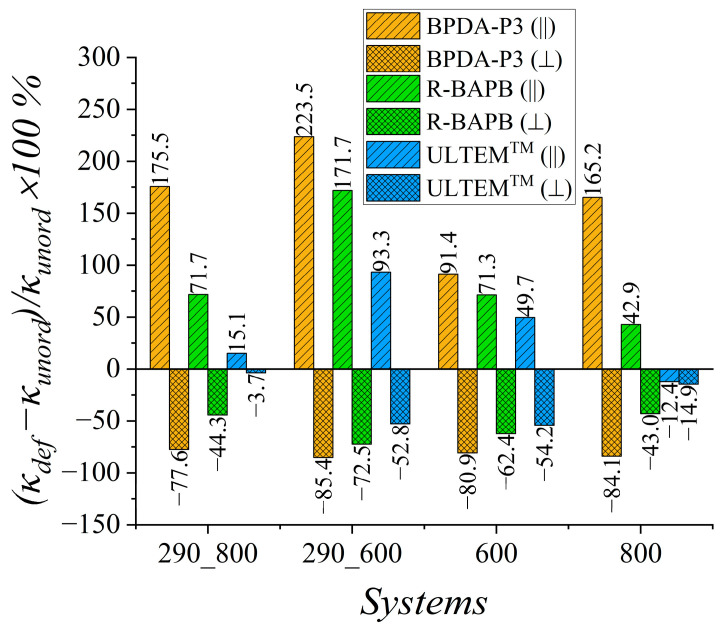
Relative percentage deviation $\left(\kappa_{def}-\kappa_{unord}\right)/\kappa_{unord}\times 100\%$ of the thermal conductivity coefficient *κ_def_* values of the deformed sample from the *κ_unord_* values for unordered samples of BPDA-P3, R-BAPB, and ULTEM^TM^ at different temperatures. Systems ‘290_800’ and ‘290_600’ were simulated at *T* = 290 K. These samples were created by cooling them from *T* = 800 K to *T* = 290 K (‘290_800’) and from *T* = 600 K to *T* = 290 K (‘290_600’). System ‘600’ was simulated at *T* = 600 K, and system ‘800’ was simulated at *T* = 800 K.

To quantitatively estimate the anisotropy of the thermal conductivity coefficient of the deformed polyimide samples, we calculated the relative percentage deviation of the *κ_def_* value for the deformed samples from the *κ_unord_* value for unordered samples of considered PIs in different directions relative to the direction of elongation (Figure 6).

Concerning the deformed samples, the relative percentage deviation was evaluated in the directions parallel (||) and perpendicular (⊥) to the deformation direction. Both semicrystalline and amorphous PIs showed a significant increase in the *κ* value for all glassy PIs in the deformation direction compared to the *κ* value of unordered samples (Figure 5 and Figure 6). However, for the semicrystalline PIs BPDA-P3 and R-BAPB, the enhancement in thermal conductivity is much more essential than that of the amorphous ULTEM^TM^. It is worth mentioning that if we compare the increase in the thermal conductivity coefficient in the deformation direction with the enhancement in the average thermal conductivity coefficient value, one can see a much higher increase along the deformation direction than along all three directions in average. The relative percentage deviation of PI BPDA-P3 along the deformation direction was 223.5% (Figure 6), whereas the relative percentage deviation for the average thermal conductivity coefficient was lower and equals only to 39.2% (Figure 4).

In turn, in the perpendicular direction, the reduction in the size of the samples during deformation impaired the thermal conductivity. Similar results have proven that thermal conductivity anisotropy has been found for other polymers [42,45,46,53,55]. The improvement [41,45] of the thermal conductivity coefficient in the parallel direction and decrease in the *κ* value of PE [45,52,54], PS [54], and PI [44] in the lateral direction have been shown. An increase of more than 160% in the deformation direction was found for amorphous PE by the elongation of polymer systems by up to 400%.

Figure 6 shows that the *κ* value in the perpendicular direction decreased from 40% to 90% for the semicrystalline polyimides BPDA-P3 and R-BAPB, as well as a slightly weaker decrease from 4% to 55% for the amorphous polyimide ULTEM^TM^.

An analysis of the influence of uniaxial deformation on *κ* values of semicrystalline and amorphous PIs showed that the heat transfer properties of both types of PIs could be significantly modified by applying uniaxial deformation. Although the average enhancement in the *κ* value of the deformed samples for semicrystalline PIs at room temperature was close to 40%, the increase in the *κ* value in the direction parallel to the deformation was much higher than that in the perpendicular direction. In the direction parallel to the deformation, the enhancement in the thermal conductivity coefficient *κ* reached approximately 223.5% for semicrystalline PI and 93.3% for amorphous PI.

### 3.3. Comparison of the Influence of Polymer Ordering after Mechanical Elongation and Self-Ordering during Long Simulation on the Thermal Conductivity Coefficient of Semicrystalline Polyimide BPDA-P3

In this section, we discuss the effect of the difference of structural ordering on the thermal conductivity coefficient of semicrystalline polymer chains [107,108,109]. To investigate the effect of the difference of structural ordering on the thermal conductivity properties of semicrystalline PIs, we compared only the *κ* value of BPDA-P3 samples ordered as follows: (i) by application of uniaxial elongation to unordered BPDA-P3 samples and (ii) BPDA-P3 samples self-ordered during 30 μs long simulations [66].

Our previous study [66] examined the transport properties of BPDA-P3, a semicrystalline PI, during the unfolding and stretching of polymer chains. This ordering was initiated during tens of microseconds of simulations at temperatures slightly above the melting point of the PI BPDA-P3 (*T_m_* = 577 K). After 30 µs of computer simulations, when the nematic order reached a value close to 0.97–0.98 [66], the initial configurations of the BPDA-P3 samples were compared with those of the mechanically stretched ones. In the present study, the systems that self-ordered during 30 μs long molecular dynamics were cooled down similarly from *T* = 600 K and *T* = 800 K to 290 K with a cooling rate *γ_c_* = 1.5 × 10^11^ K/min. The temperature dependence of the density of the analyzed samples ordered during uniaxial elongation and self-ordered during a 30 μs long simulation is shown in Figure S8 in the Supplementary Materials.

**Figure 7:**
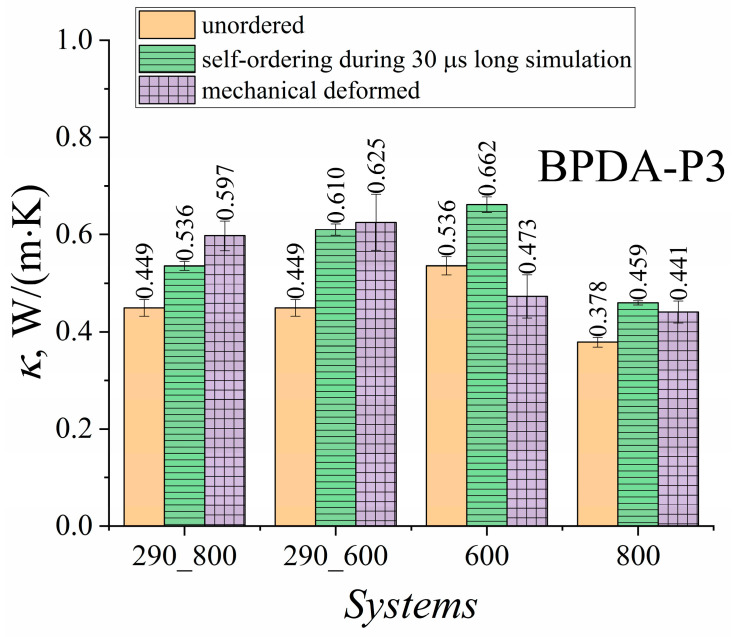
BPDA-P3 thermal conductivity coefficient *κ* of unordered, deformed, and self-ordered samples during a 30 µs long simulation of PI BPDA-P3. Systems ‘290_800’ and ‘290_600’ were simulated at *T* = 290 K. These samples were created by cooling them from *T* = 800 K to *T* = 290 K (‘290_800’) and from *T* = 600 K to *T* = 290 K (‘290_600’). System ‘600’ was simulated at *T* = 600 K, and system ‘800’ was simulated at *T* = 800 K.

The ordering of the BPDA-P3 samples led to the densification of the systems. Both ordered samples that were cooled, starting from *T* = 800 K and *T* = 600 K, had very close temperature dependencies of the density. However, cooling from 600 K to room temperature led to much higher density of the PI samples. The *κ* values were calculated at 290, 600, and 800 K, as shown in Figure 7.

As expected, both samples ordered by mechanical deformation and self-ordered during a 30 µs long simulation demonstrated an increase in the *κ* value at room temperature (*T* = 290 K). At *T* = 290 K, the samples cooled from *T* = 800 K showed a higher thermal conductivity coefficient in the case of mechanical deformation than the self-ordered samples during long simulations. Cooling from *T* = 600 K led to an increase in the *κ* value in the glassy state because of the much stronger preservation of the ordered structure of PI BPDA-P3. The preserved ordering of the polymer chains, which in the end greatly improves the heat transfer, compared to the situation when the structurally ordered samples cooled from *T* = 800 K correspond to a highly mobile melt. Although the samples were cooled quite rapidly compared to the experimental cooling rate, the instance configurations of the ordered samples apparently did not undergo substantial derangement of the polymer chains, and the *κ* value remained higher than that of the undeformed samples. At higher temperatures, when the temperature was 600 K or 800 K, the self-ordering during the 30 µs long simulation showed a greater increase in *κ* value compared to the mechanically elongated samples.

**Figure 8:**
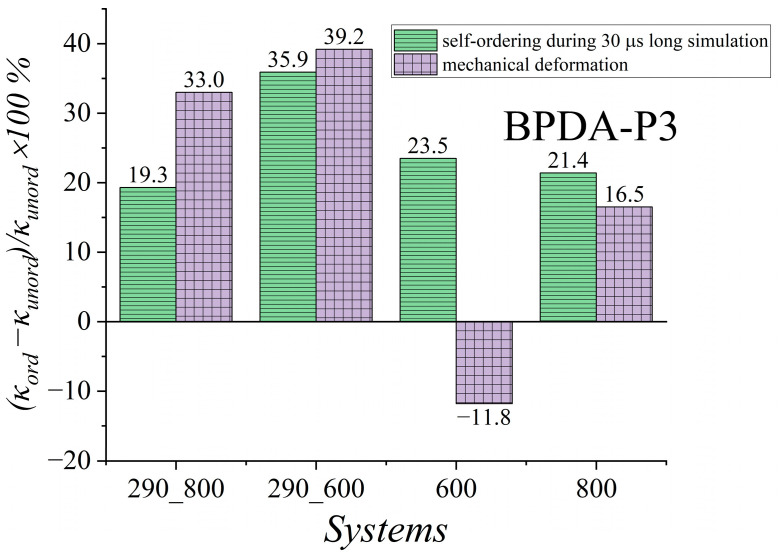
Relative percentage deviation $\left(\kappa_{ord}-\kappa_{unord}\right)/\kappa_{unord}\times 100\%$ of thermal conductivity coefficient *κ_ord_* value for ordered samples from *κ_unord_* value for unordered samples of BPDA-P3 calculated at different temperatures. Systems ‘290_800’ and ‘290_600’ were simulated at *T* = 290 K. These samples were created by cooling them from *T* = 800 K to *T* = 290 K (‘290_800’) and from *T* = 600 K to *T* = 290 K (‘290_600’). System ‘600’ was simulated at *T* = 600 K, and system ‘800’ was simulated at *T* = 800 K.

To analyze how the thermal conductivity coefficient *κ* is enhanced in terms of quantity, we calculated the relative percentage deviation $\frac{\left(\kappa_{ord}-\kappa_{unord}\right)}{\kappa_{unord}}\times 100\%$ of the thermal conductivity coefficient *κ_ord_* value for the ordered samples from that for the unordered ones’ *κ_unord_* value, as shown in Figure 8.

Analysis of the results in Figure 8 shows that the enhancement in *κ* values of BPDA-P3 for both ordering cases at room temperature when the samples were cooled from *T* = 600 K was rather close, and did not exceed 40%. However, when cooling was carried out from *T* = 800 K, the BPDA-P3 samples self-ordered during the long simulation showed a smaller improvement in the heat transfer properties, whereas the thermal conductivity properties of the mechanically ordered samples had a weaker deterioration. This might be because the deformation was carried out at a temperature close to the melting point of BPDA-P3. The deformation of the PI sample at a temperature close to the transition temperature may cause heat flux fluctuations that are combined with a decrease in the *κ* value. It is worth noting that at *T* = 600 K, the mechanically ordered samples demonstrated a decrease in *κ* value compared to the self-ordered samples over a 30 μs long simulation. When the temperature increased to 800 K, the heat transfer properties of both ordered systems increased by approximately 21.4% and 16.5%, respectively. The enhancement in the *κ* value might be related not only to the emergence of order in polymer chains, but also to an increase in the density of ordered systems [110].

Overall, both types of oriented systems exhibited a rather similar increase in *κ* value in the glassy state after fast cooling. The maximum enhancement in the thermal conductivity properties did not exceed 40% for either case when the cooling started at *T* = 600 K. However, when cooling started at *T* = 800 K, the mechanical deformation of the semicrystalline PI BPDA-P3 resulted in a more significant enhancement in the value of the thermal conductivity coefficient *κ* compared to *κ* value of self-ordered systems during a 30 μs long simulation due to the arbitrarily oriented polymer chains relative to different coordinate axes. With an increase in temperature (*T* = 600 K and *T* = 800 K) greater than *T_g_*, the *κ* value of the ordered samples showed a slight decrease in enhancement compared to the *κ* value of the ordered sample in a glassy state.

## 4. Conclusions

The development of new thermal interface materials that combine necessary dielectric properties with improved conductive properties is an important industrial task. To provide an overview of this problem, microsecond-scale computer simulations were performed to investigate the thermal conductivity of TIMs based on three thermoplastic PIs. The influence of mechanical deformation performed in melting state on the thermal conductivity coefficient *κ* of three thermostable PI including two semicrystalline BPDA-P3 and R-BAPB as well as amorphous ULTEM^TM^ was considered at different temperatures.

To study the thermal conductivity properties of the thermoplastic polyimides, the choice of the partial charge calculation method for their model based on the GAFF force field as well as the choice of thermal conductivity coefficient calculation method were performed. The best agreement with thermophysical properties of considered PIs was obtained for the HF/6-31G* (RESP) method using EMD.

The influence of mechanical deformation of polymer samples on the thermal conductivity coefficient of semicrystalline and amorphous polyimides was studied. The samples stretched up to 200% of their initial size, demonstrating the initiation of nematic ordering of the polymer chains along the deformation direction. Semicrystalline PIs were ordered upon deformation, which was significantly more substantial than that of the amorphous PIs. The enhancement in the thermal conductivity of the ordered PI samples accounts for the appearance of structural ordering in one direction in the polyimide chains. The anisotropy of the heat transfer properties was found: the thermal conductivity coefficient along the deformation was essentially higher than that computed in the lateral direction.

The impact of two different structural orderings on the thermal conductivity coefficient of PI chains was studied. With regard to the semicrystalline PI BPDA-P3, the thermal conductive properties of the samples ordered over tens of microseconds by computer simulations were compared with the thermal conductive properties of the samples ordered by mechanical elongation. When cooling started from *T* = 800 K, a higher enhancement in the thermal conductive properties of the samples ordered by mechanical elongation was observed compared to the properties of the unordered samples. However, when cooling started at 600 K, the results reveal that both structural orderings caused a similar enhancement in thermal conductivity in the glassy state. For this case, the EMD method showed an almost 40% increase in the thermal conductivity coefficient of semicrystalline PI. This might be useful for the creation of the BPDA-P3 samples by applying mechanical elongation, which would reduce the computational time for the creation of BPDA-P3 self-ordered 30 µs long computer simulation samples.

## Figures and Tables

**Table 1:** The thermal conductivity coefficient values *κ* and the relative percentage deviation $\left(\kappa^{sim}-\kappa^{exp}\right)/\kappa^{exp}\times 100\%$ of simulated thermal conductivity coefficient values $\kappa^{sim}$ from the experimental thermal conductivity coefficient value $\kappa^{exp}$ of PI ULTEM^TM^ at 290 K were calculated by different thermal conductivity coefficient calculation methods (EMD or NEMD). For the considered systems, partial charges were computed using different partial charge calculation methods (AM1-BCC or HF/6-31G* (RESP)).

Partial Charge Calculation Method	AM1-BCC		HF/6-31G* (RESP)	
Thermal conductivity calculation methods	EMD	NEMD	EMD	NEMD
Thermal conductivity of ULTEM^TM^, *T* = 290 K	0.284 ± 0.005	0.296 ± 0.001	0.265 ± 0.001	0.339 ± 0.001
$\left(\kappa^{sim}-\kappa^{exp}\right)/\kappa^{exp}\times 100\%$	29.1%	34.5%	20.5%	54.1%

## Data Availability

The data presented in this study are available upon request from the corresponding author.

## Supplementary Materials

The following supporting information can be downloaded at: https://www.mdpi.com/article/10.3390/polym15132926/s1. Figure S1. Radius of gyration of polymer chains of different polyimides: (a) BPDA-P3, (b) R-BAPB, and (c) ULTEM^TM^ as a function of time when partial charges were calculated using the AM1-BCC and HF/6-31G* (RESP) partial charge calculation methods. Figure S2. Mean squared displacement of the center-of-mass of the polyimide chain of BPDA-P3, R-BAPB, and ULTEM^TM^ when partial charges were calculated using the partial charge calculation methods (a,c) AM1-BCC and (b,d) HF/6-31G* (RESP). Figure S3. Time dependence of the nematic order parameter *S_N_* for (a) BPDA-P3, (b) R-BAPB, and (c) ULTEM^TM^ polyimides. Figure S4. Mass density of the BPDA-P3, R-BAPB, and ULTEM^TM^ polyimides as a function of temperature for the samples when the partial charges were calculated using the (a) AM1-BCC and (b) HF/6-31G* (RESP) methods. Table S1. The glass transition temperatures (*T_g_*) of the studied PIs when electrostatic calculation were parameterized using two different partial charge calculation methods, AM1-BCC and HF/6-31G* (RESP). Figure S5. Coefficient of thermal expansion (*CTE*) of the BPDA-P3, R-BAPB, and ULTEM^TM^ polyimides as a function of temperature for the samples when partial charges were calculated by (a) AM1-BCC or (b) HF/6-31G* (RESP) partial charge calculation methods. Table S2. *CTE* values of the PIs calculated for the systems when partial charges were computed using the two partial charge calculation methods, AM1-BCC or HF/6-31G* (RESP). Figure S6. Thermal conductivity coefficients *κ* of undeformed BPDA-P3, R-BAPB, and ULTEM^TM^ at different temperatures (290, 600, and 800 K). Figure S7. Phonon vibrational density of states (*VDOS*) of an undeformed sample of BPDA-P3, R-BAPB, and ULTEM^TM^ atoms at *T* = 290 K. Figure S8. Mass density *ρ* of the unordered, deformed and self-ordered during a 30 µs long simulation samples of polyimide BPDA-P3 as a function of temperature when cooling is performed from *T* = 800 K to *T* = 290 K or from *T* = 600 K to *T* = 290 K.
