# Supplementary material for: The Effect of Mechanical Elongation on the Thermal Conductivity of Amorphous and Semicrystalline Thermoplastic Polyimides: Atomistic Simulations
Source: Polymers (Basel). 2023 Jul 1;15(13):2926. doi: 10.3390/polym15132926 (PMC10346438; doi:10.3390/polym15132926)
Supplement: Supplementary file 1 [file polymers-15-02926-s001.zip › polymers-2454972-supplementary.pdf]

Supplementary Materials

The Effect of Mechanical Elongation on the Thermal Conductivity of Amorphous and Semicrystalline Thermoplastic Polyimides: Atomistic Simulations

Victor M. Nazarychev * and Sergey V. Lyulin

Institute of Macromolecular Compounds, Russian Academy of Sciences, Bolshoi Prospect V.O. 31, St. Petersburg 199004, Russia; s.v.lyulin@gmail.com
* Correspondence: nazarychev@imc.macro.ru; Tel.: +7-(812)-3230216

1. Average radius of gyration of studied polyimides.

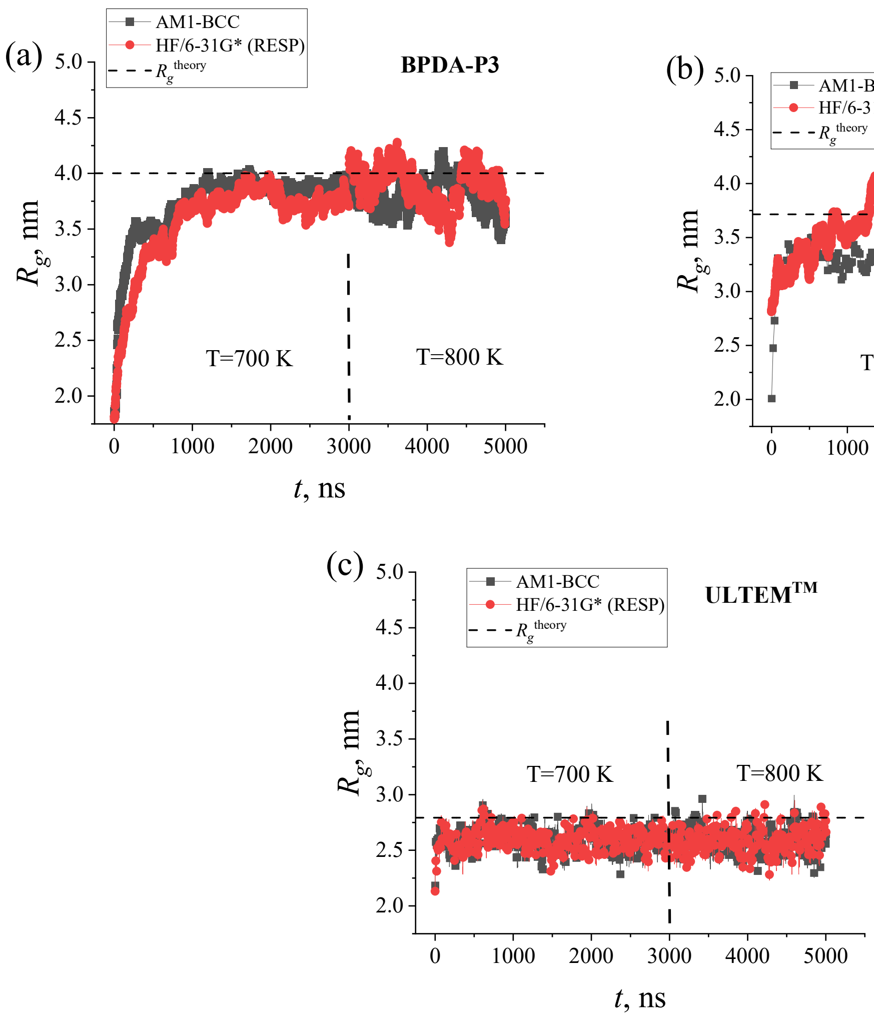

Figure S1. Radius of gyration of polymer chains of different polyimides: a) BPDA-P3, b) R-BAPB, and c) ULTEM™ as a function of time when partial charges were calculated using the AM1-BCC and HF/6-31G* (RESP) partial charge calculation methods. The dashed horizontal lines correspond to the radius of gyration predicted analytically for the free joint model.

2. Mean squared displacement of the center of mass of the studied polyimides.

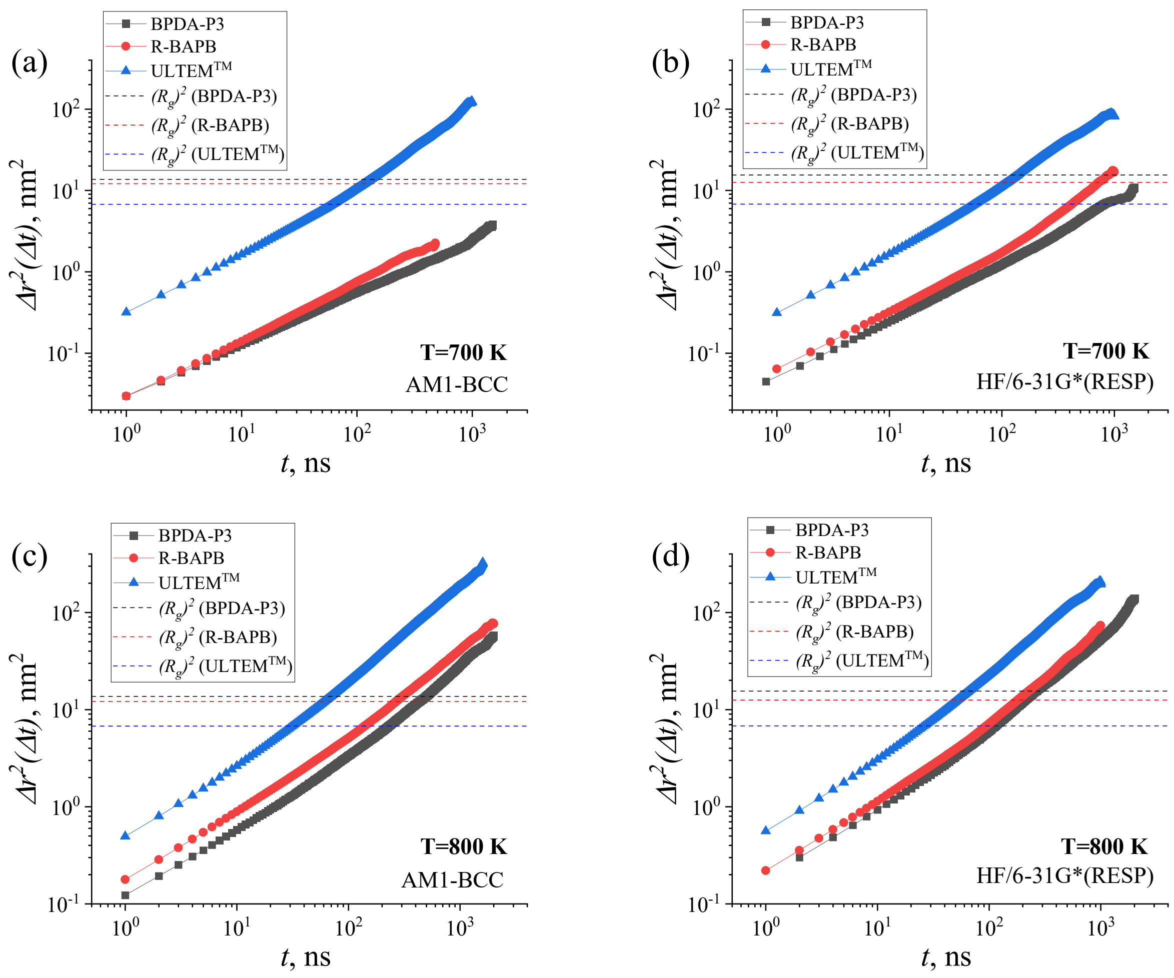

Figure S2. Mean squared displacement of the center-of-mass of the polyimide chain of BPDA-P3, R-BAPB, and ULTEM™ when partial charges were calculated using the partial charge calculation methods (a, c) AM1-BCC and (b, d) HF/6-31G* (RESP). The temperature is (a, b) 700 K and (c, d) 800 K. The dashed lines indicate that the mean square displacement is equal to the square of the average radius of gyration of the considered PIs.

3. Temperature dependence of the nematic order parameter of the studied polyimides. The deformed samples.

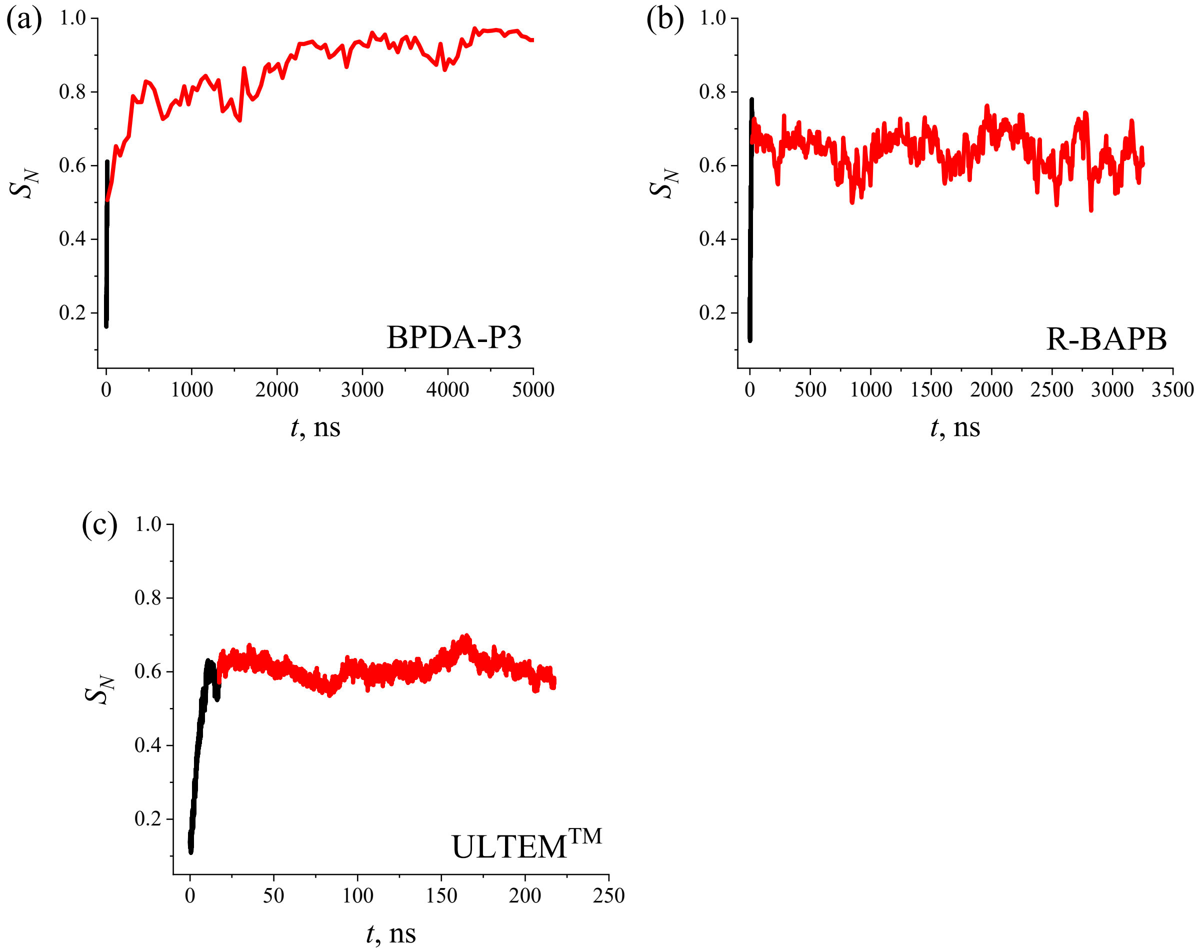

Figure S3. Time dependence of the nematic order parameter S_N for (a) BPDA-P3, (b) R-BAPB, and (c) ULTEM^{TM} polyimides. The change in the time dependence of the nematic order parameter during deformation is shown by the black curve, and the change in the time dependence of the nematic order parameter during the additional simulation after deformation is shown by the red curve. The temperature is 600 K.

4. Procedure for changing the polyimide models based on the Gromos53a5 force field on models based on the GAFF force field.

In the beginning, the parameters describing the intramolecular and intermolecular interactions of the samples in the polyimide model based on the Gromos53a5 force field were changed to the functions and parameter values used in the GAFF force field, saving all the coordinates of the atoms. Then, a minimum energy search was carried out using the steepest descent algorithm for 50000 simulation steps until the maximum force F_{max} was less than 10 kJ/(mol·nm).

Subsequently, we ran two rather short simulations (during 1 ns): (i) in the *NVT* ensemble with a simulation step equal to 1 fs and (ii) in the *NPT* ensemble with the same parameters. For the relaxation of the samples in the *NVT* and *NPT* ensembles, the Berendsen thermostat and barostat were used with time relaxation constants equal to 0.1 and 1 ps, respectively. After, the simulation step was doubled from 1 to 2 fs and a simulation of 1 ns was performed. The force field change procedure was repeated for the two PIs BPDA-P3 and R-BAPB.

5. **Temperature dependence of density of the studied polyimides. Calculation of glass transition temperature and coefficient of thermal expansion.**

To determine the glass transition temperature, the density-temperature relationship for the three PIs was analyzed. Calculations were performed for the samples for which electrostatic interactions were parameterized using the AM1-BCC and HF/6-31G* (RESP) methods. The temperature dependence of the density has two parts that are close to linear dependence in the high- and low-temperature domains. The glass transition temperature was calculated as the temperature corresponding to the intersection of the fitting functions of these parts (Figure S4).

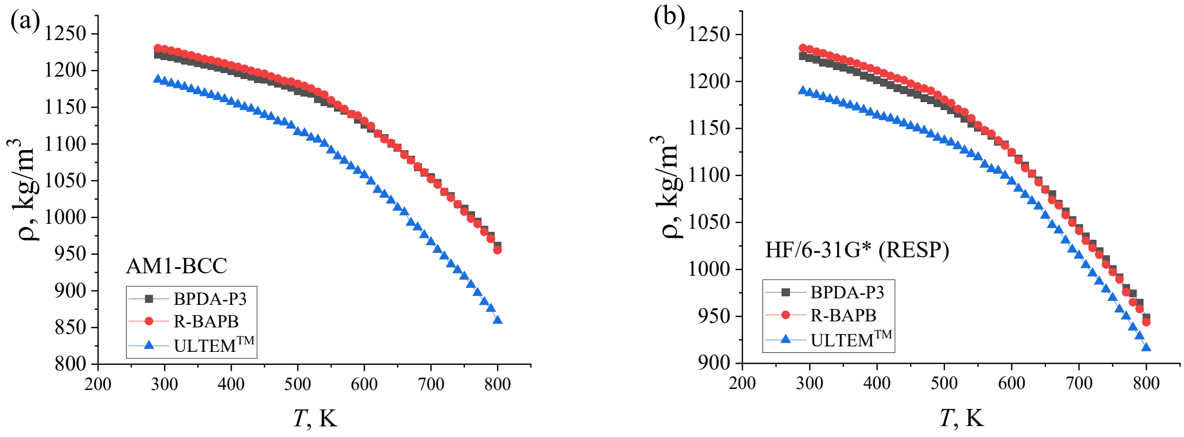

Figure S4. Mass density of the BPDA-P3, R-BAPB, and ULTEM™ polyimides as a function of temperature for the samples when the partial charges were calculated using the (a) AM1-BCC and (b) HF/6-31G* (RESP) methods. The glass transition temperature was computed using two temperature intervals: $T = 300$ K to $T = 400$ K and $T = 700$ K to $T = 790$ K.

Although there was a significant difference in the cooling rate between the experimental and computer simulation procedures, it was possible to qualitatively predict the experimental ratio between the T_g values of the PIs in Table S1 through the simulation.

Table S1. The glass transition temperatures (T_g) of the studied PIs when electrostatic interactions were parameterized using two different partial charge calculation methods,

AM1-BCC and HF/6-31G* (RESP), as well as the experimentally measured T_g values. The standard deviation of T_g did not exceed 5 K.

Systems	Polyimide		
	BPDA-P3	R-BAPB	ULTEM™
AM1-BCC	577 K	571 K	550 K
HF/6-31G* (RESP)	568 K	553 K	589 K
Experiment	487 K [1]	478 [2]	490 K [3]

The calculated T_g values were significantly dependent on the methodology of the partial charge calculations. The results of the cooling procedure led to the following conclusions. The *ab initio* partial charge calculation method HF/6-31G*(RESP) is much better than the semi-empirical method AM1-BCC. First, only the HF/6-31G*(RESP) method allows reproducing the experimental ratio between the T_g values of three considered PIs: $T_g^{ULTEM^{TM}} > T_g^{BPDA-} > T_g^{R-BAPB}$. Unlike the experimental findings, when AM1-BCC partial charges were considered, the T_g value of ULTEM™ was lower than that of BPDA-P3 and R-BAPB.

As demonstrated in a previous study [4], the *CTE* value in the glassy state is a thermophysical characteristic that remains almost unchanged despite the difference in the cooling rates between the experimental and simulated conditions. The value of the thermal expansion coefficient *CTE* was calculated using the temperature dependence of density as $CTE = 1/V_0 \frac{dV}{dT}$, where $\frac{dV}{dT}$ is the temperature gradient and V_0 is the volume at T = 300 K. The mean *CTE* values were calculated by averaging the *CTE* values in the temperature range from T = 300 K to T = 400 K, Figure S5.

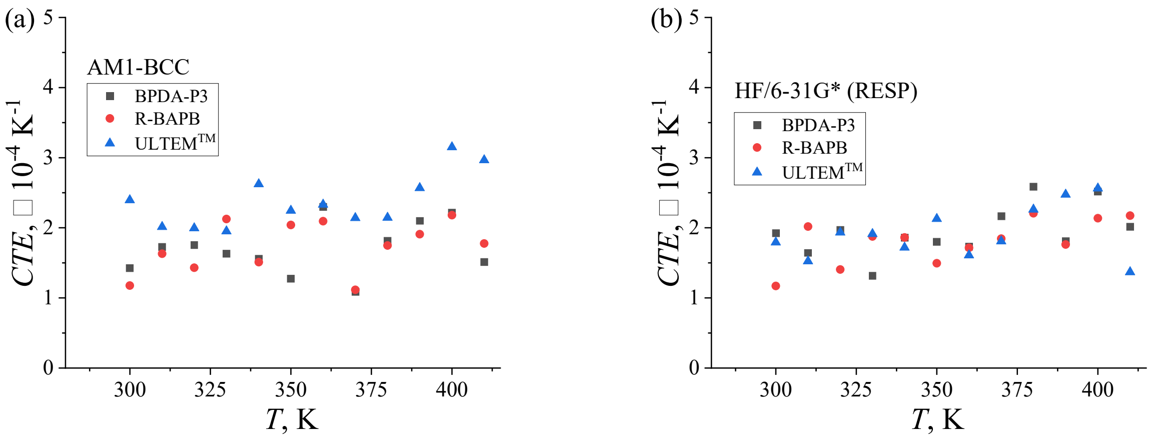

Figure S5. Coefficient of thermal expansion (*CTE*) of the BPDA-P3, R-BAPB, and ULTEM™ polyimides as a function of temperature when electrostatic interactions were parameterized using two different partial charge calculation methods: (a) AM1-BCC or (b) HF/6-31G* (RESP).

The average *CTE* values for the glassy state are listed in Table S2.

Table S2. The *CTE* values of the PIs were calculated for the systems when partial charges were computed using two partial charge calculation methods, AM1-BCC or HF/6-31G* (RESP), and the *CTE* values were experimentally measured.

Systems	Polyimide		
	BPDA-P3	R-BAPB	ULTEM™
AM1-BCC	1.7 ± 0.37	1.73 ± 0.36	2.38 ± 0.39
HF/6-31G* (RESP)	1.95 ± 0.35	1.81 ± 0.32	1.92 ± 0.37
Experiment	-	1.60 K^{-1} [2]	1.65 K^{-1} [3]

Table S2 shows that when comparing HF/6-31G* (RESP) with AM1-BCC, the results of system when partial charges were computed by the *ab initio* quantum chemistry technique agreed well with the experimental results. Within the margin of error, the *CTEs* of PI R-BAPB and ULTEM™ were substantially closer to the experimental *CTE* value when using the partial charge calculation method HF/6-31G*(RESP).

In essence, the AM1-BCC procedure increased the *CTE* value of polyimide ULTEM™. The HF/6-31G* (RESP) approach provides the most accurate predictions of the thermal-physical characteristics, as shown by the comparison of T_g and *CTE* values.

6. Influence of the difference in the chemical structure of polyimides on their thermal conductivity coefficients.

Increasing the temperature from 290 to 600 K led to a small increase in the thermal conductivity of all the studied PIs. A further rise in temperature beyond T_g to 800 K resulted in a more noticeable reduction in κ value. Figure S6 illustrates that an increase in temperature to T = 800 K decreases κ value relative to that at T = 600 K. In general, the thermal conductivity of the undeformed samples did not significantly depend on the temperature.

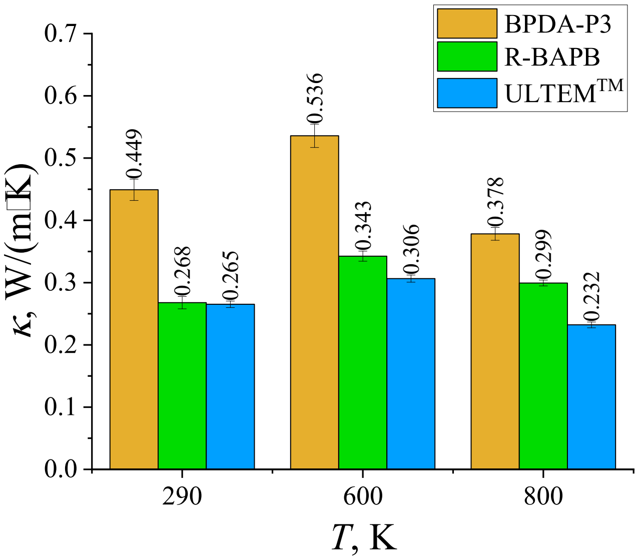

Figure S6. Thermal conductivity coefficients κ of undeformed BPDA-P3, R-BAPB, and ULTEM™ at different temperatures (290, 600, and 800 K).

For the considered EMD method for the thermal conductivity coefficient calculation, the κ values for the considered PIs are related as $\kappa^{BPDA-} > \kappa^{R-BA} > \kappa^{ULTE\quad TM}$. The observed ratio of κ values may be attributed to variations in the flexibility of the repeating units of PI, resulting in differences in their spatial arrangement and consequent variations in the distribution of the free volume within the bulk. ULTEM™ exhibits a shorter phonon free-path length than semi-crystalline BPDA-P3 and R-BAPB because of the presence of

a CH_3-C-CH_3 (dimethyl) group in its dianhydride fragment, which leads to an increase in the free volume of the polymer chain.

We determined the phonon vibrational density of states (*VDOS*) to understand the effect of differences in chemical structure on phonon transport in polymer materials.

$$VDOS(\omega) = \int_{-\infty}^{\infty} VACF(t) \cdot \exp(-2\pi i \omega t)\, dt, \quad \textbf{(S1)}$$

$$VACF(t) = \frac{\sum_{i=1}^{N} \langle v_i(t) \cdot v_i(0) \rangle}{\sum_{i=1}^{N} \langle v_i(0) \cdot v_i(0) \rangle}, \quad \textbf{(S2)}$$

where v_i is the velocity of the atoms, N is the number of atoms, and *VACF(t)* is the normalized velocity autocorrelation function, as shown in Figure S7.

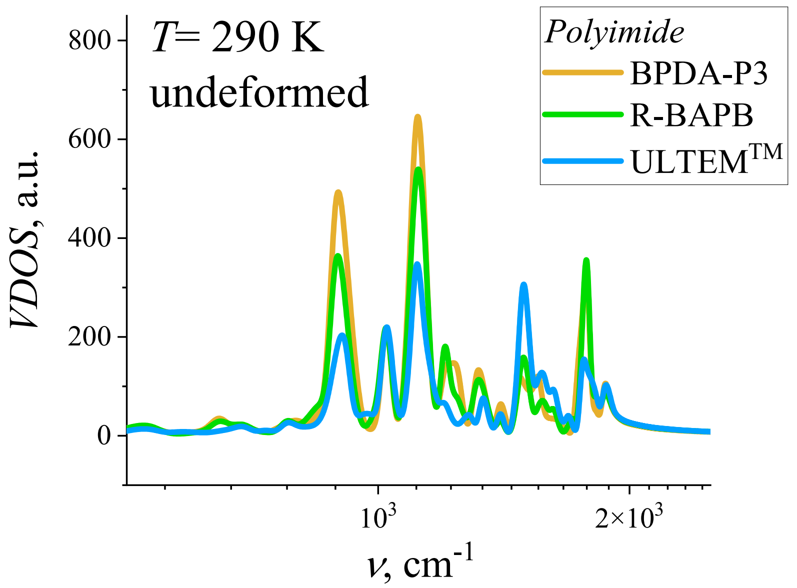

Figure S7. Phonon vibrational density of states (*VDOS*) of an undeformed sample of BPDA-P3, R-BAPB, and ULTEM™ atoms, T = 290 K.

The vibrational density of states (*VDOS*) of the PIs had distinct peaks at ν equals 890 cm^{-1} and ν equals 1100 cm^{-1}, whose ratio of amplitude could be related to the ratio of the

thermal conductivity coefficients $\kappa^{BPDA-P3} > \kappa^{R-BAP} > \kappa^{ULTEM^{TM}}$. The *VDOS* of BPDA-P3 exhibited the most prominent peaks at the selected frequencies, whereas ULTEM^{TM} exhibited the least pronounced *VDOS* (Figure S7).

7. Temperature dependence of the density of semicrystalline polyimide BPDA-P3.

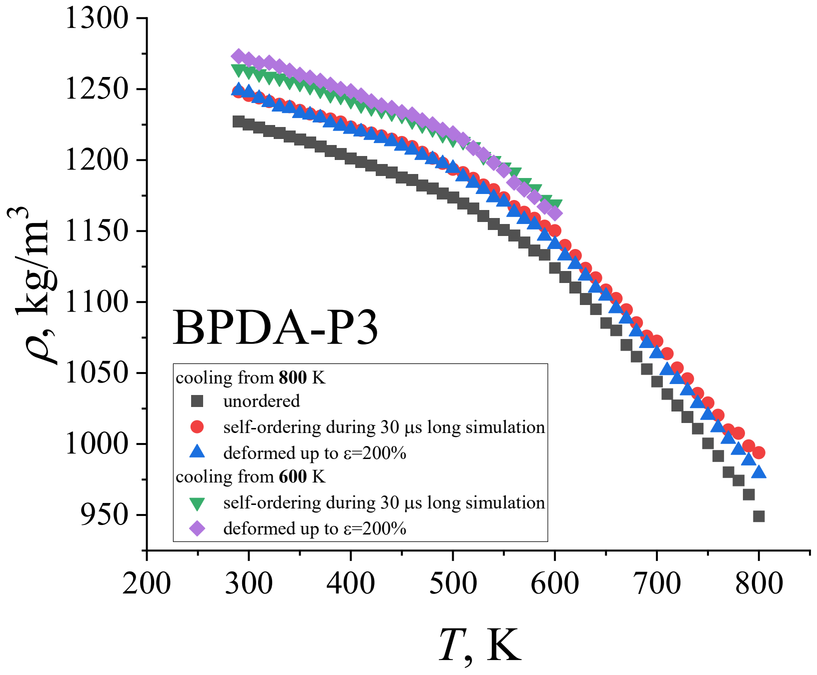

Figure S8. Mass density ρ of the unordered, deformed and self-ordered during a 30μs-long simulation samples of polyimide BPDA-P3 during a 30μs-long simulation as a function of temperature when cooling is performed from T = 800 K to T = 290 K or from T = 600 K to T = 290 K.

References.

1. Hegde, M.; Lafont, U.; Norder, B.; Samulski, E. T.; Rubinstein, M.; Dingemans, T. J. SWCNT induced crystallization in amorphous and semi-crystalline poly(etherimide)s: Morphology and thermo-mechanical properties. *Polymer* **2014**, *55*, 3746–3757, doi:10.1016/j.polymer.2014.06.017.

2. Nazarychev, V. M.; Larin, S. V.; Yakimansky, A. V.; Lukasheva, N. V.; Gurtovenko, A. A.; Gofman, I. V.; Yudin, V. E.; Svetlichnyi, V. M.; Kenny, J. M.; Lyulin, S. V. Parameterization of electrostatic interactions for molecular dynamics simulations of heterocyclic polymers. *Journal of Polymer Science Part B: Polymer Physics* **2015**, *53*, 912–923, doi:10.1002/polb.23715.

3. Falkovich, S. G.; Lyulin, S. V.; Nazarychev, V. M.; Larin, S. V.; Gurtovenko, A. A.; Lukasheva, N. V.; Lyulin, A. V. Influence of the electrostatic interactions on thermophysical properties of polyimides: Molecular-dynamics simulations. *Journal of Polymer Science Part B: Polymer Physics* **2014**, *52*, 640–646, doi:10.1002/polb.23460.

4. Lyulin, S. V.; Larin, S. V.; Gurtovenko, A. A.; Nazarychev, V. M.; Falkovich, S. G.; Yudin, V. E.; Svetlichnyi, V. M.; Gofman, I. V.; Lyulin, A. V. Thermal properties of bulk polyimides: insights from computer modeling versus experiment. *Soft matter* **2014**, *10*, 1224–32, doi:10.1039/c3sm52521j.
